# Targeted and novel therapy in advanced gastric cancer

**DOI:** 10.1186/s40164-019-0149-6

**Published:** 2019-10-11

**Authors:** Julie H. Selim, Shagufta Shaheen, Wei-Chun Sheu, Chung-Tsen Hsueh

**Affiliations:** 1 School of Pharmacy, Loma Linda University, Loma Linda, CA 92350 USA; 2 Division of Oncology, Stanford Cancer Center, Stanford, CA 94304 USA; 3 Department of Internal Medicine, Richmond University Medical Center, Staten Island, NY 10310 USA; 4 Division of Medical Oncology and Hematology, Department of Medicine, Loma Linda University, 11175 Campus Street, CSP 11015, Loma Linda, CA 92354 USA

**Keywords:** Gastric cancer, Targeted therapy, Human epidermal growth factor receptor 2, Programmed death-1, Vascular endothelial growth factor receptor 2

## Abstract

The systemic treatment options for advanced gastric cancer (GC) have evolved rapidly in recent years. We have reviewed the recent data of clinical trial incorporating targeted agents, including inhibitors of angiogenesis, human epidermal growth factor receptor 2 (HER2), mesenchymal–epithelial transition, epidermal growth factor receptor, mammalian target of rapamycin, claudin-18.2, programmed death-1 and DNA. Addition of trastuzumab to platinum-based chemotherapy has become standard of care as front-line therapy in advanced GC overexpressing HER2. In the second-line setting, ramucirumab with paclitaxel significantly improves overall survival compared to paclitaxel alone. For patients with refractory disease, apatinib, nivolumab, ramucirumab and TAS-102 have demonstrated single-agent activity with improved overall survival compared to placebo alone. Pembrolizumab has demonstrated more than 50% response rate in microsatellite instability-high tumors, 15% response rate in tumors expressing programmed death ligand 1, and non-inferior outcome in first-line treatment compared to chemotherapy. This review summarizes the current state and progress of research on targeted therapy for advanced GC.

## Background

Gastric cancer (GC), including adenocarcinoma of the gastroesophageal junction (GEJ) and stomach, is the fifth most common cancer and the third leading cause of cancer death [1]. There is a higher incidence of GC in Eastern Europe, Eastern Asia and South America. The survival of advanced GC is poor with 5-year survival rate of less than 10% [2]. Cytotoxic chemotherapy remains the backbone of systemic treatment; platinum- and fluoropyrimidine-based combination therapy is used as the preferred first-line treatment of advanced GC. After first-line therapy, randomized trials of single-agent cytotoxic chemotherapy such irinotecan, paclitaxel and docetaxel have demonstrated improved overall survival (OS) and quality of life when compared with best supportive care [3]. In USA, there has been a significant reduction in inpatient GC mortality which is consistent with overall decrease in GC-related deaths [4].

There have been several efforts to perform large-scale molecular profiling and classification of GC. Lei et al. compared gene expression patterns among 248 GC from Singaporean patients and identified 3 major subtypes: proliferative (characterized by high genomic instability, TP53 mutations and DNA hypomethylation), metabolic [more sensitive to 5-fluorouracil (5-FU) than other subtypes], and mesenchymal [with features of cancer stem cells; cell lines of this subtype particularly sensitive to inhibitors of phosphatidylinositol-3-kinase/mammalian target of rapamycin (PI3K/mTOR) pathway] [5]. The Cancer Genome Atlas (TCGA) Research Network characterized four molecular subtypes of GC by six molecular biology approaches: exome sequencing analysis, copy number variation analysis, DNA methylation profile, micro-RNA sequencing, mRNA sequencing and reverse phase protein array [6]. Using primary GC tumor tissue from 295 predominantly Caucasian patients not treated with prior chemotherapy or radiotherapy, the TCGA study showed about 9% with Epstein–Barr virus (EBV) infection associated with frequent phosphatidylinositol-4,5-bisphosphate 3-kinase catalytic subunit alpha (PIK3CA) mutation, programmed death ligand 1/2 (PD-L1/PD-L2) overexpression and the best prognosis; 21% with microsatellite instability (MSI) associated with increased tumor mutation burden; 50% with chromosomal instability (CIN) associated with frequent amplifications of amplifications of vascular endothelial growth factor A (VEGFA) and receptor tyrosine kinase (RTK) genes such as human epidermal growth factor receptor 2 (HER2) as well as mutation of TP53; 20% genomically stable (GS) associated with frequent mutations in motility and adhesion genes as well as the worst prognosis [7]. The subsequent combined analysis of esophageal cancer and GC showed most of esophageal and GEJ adenocarcinoma were classified as CIN [8].

In 2015, the Asian Cancer Research Group used array-based gene expression profiling on 300 primary GC tumor specimens at the time of total or subtotal gastrectomy from Samsung Medical Center in Korea to identify four molecular subtypes that were associated with survival and recurrence patterns after surgery: MSI (23% of the cohort) with loss of the loss of mutL homolog 1 (MLH1) expression, microsatellite stable (MSS) with epithelial-to-mesenchymal transition (EMT) signature (15%) with loss of cadherin 1 expression, MSS with intact TP53 activity (MSS/TP53+; 26%), and MSS with loss of TP53 function (MSS/TP53−; 36%) [9]. They observed that among the 4 molecular subtypes, patients with MSS/EMT had more advanced disease and the worst prognosis with recurrence rate of 63%; on the contrary, patients with MSI had less advanced disease and the best prognosis with recurrence rate of 22%. EBV infection occurred more frequently in the MSS/TP53+ group than in the other groups. It appeared that MSS/TP53+ somewhat overlapped with EBV type, and MSS/TP53− overlapped with CIN defined by TCGA, respectively.

These classifications have provided significant understanding of molecular profiling and heterogeneity of GC among different demographics. The information from these analyses has indicated several actionable genetic alterations such as HER2 amplification (~ 15%, more commonly seen in GEJ and CIN), mesenchymal–epithelial transition (MET) amplification (~ 20%), fibroblast growth factor receptor 2 (FGFR2) amplification/mutation (~ 5–10%), PD-L1 amplification (more commonly seen in EBV infection), MSI-high (~ 15%), etc. These findings provide opportunities for personalized treatment in advanced GC.

Herein, we have reviewed the evidenced-based data from clinical trials on the use of targeted therapeutics in advanced GC including completed phase III (Table 1) and randomized phase II (Table 2) studies. These results have led to the following approved standard of care in USA: trastuzumab for patients with HER-2 positive disease in the first-line setting, monoclonal antibody against vascular endothelial growth factor receptor 2 (VEGFR2) known as ramucirumab as second- or third-line therapy, TAS-102 as third-line therapy, and programmed death-1 (PD-1) inhibitor pembrolizumab in MSI-high tumors as the second-line choice or PD-L1 expressing tumors in the third-line setting. As we are witnessing a significant change of treatment landscape, patients with advanced GC are having more treatment options available and living longer. Additionally, we have discussed ongoing phase III trials with targeted agents and the use of biomarkers for patient selection and outcome prediction with these agents (Table 3).

**Table 1:** Completed phase III trials with targeted agents in advanced gastric cancer

Targeted pathway	Agent	Trial	Selection biomarker/trial design	Overall survival benefit/months (experimental versus control)
HER2	Trastuzumab	ToGA/1st-line [13]	HER2/chemotherapy with or without trastuzumab	Positive (HR 0.74)/13.8 versus 11.1
	Trastuzumab	HELOISE/1st-line phase IIIb [14]	HER2/chemotherapy with standard-dose versus high-dose trastuzumab	Negative (HR 1.24)/12.5 versus 10.6
	Trastuzumab/pertuzumab	JACOB/1st-line [21]	HER2/trastuzumab plus chemotherapy with pertuzumab or placebo	Negative (HR 0.84)/17.5 versus 14.2
	Trastuzumab emtansine (T-DM1)	GATSBY/2nd-line [29]	HER2/T-DM1 versus taxane	Negative (HR 1.15)/7.9 versus 8.6
	Lapatinib	LoGIC/1st-line [41]	HER2/chemotherapy with lapatinib or placebo	Negative (HR 0.91)/12.2 versus 10.5
	Lapatinib	Tytan/2nd-line [43]	HER2/paclitaxel with or without lapatinib	Negative (HR 0.84)/11.0 versus 8.9
Angiogenesis	Bevacizumab	AVAGAST/1st-line [75]	None/chemotherapy with bevacizumab or placebo	Negative (HR 0.87)/12.1 versus 10.1
	Bevacizumab	AVATAR/1st-line [78]	None/chemotherapy with bevacizumab or placebo in Chinese patients	Negative (HR 1.11)/10.5 versus 11.4
	Ramucirumab	RAINFALL/1st-line [66]	HER2(−)/chemotherapy with ramucirumab or placebo	Negative (HR 0.96)/11.2 versus 10.7
	Ramucirumab	RAINBOW/2nd-line [61]	None/paclitaxel with ramucirumab or placebo	Positive (HR 0.807)/9.6 versus 7.4
	Ramucirumab	REGARD/2nd and 3rd-line [60]	None/ramucirumab versus placebo	Positive (HR 0.776)/5.2 versus 3.8
	Apatinib	NCT01512745/3rd-line [73]	None/apatinib versus placebo	Positive (HR 0.71)/6.5 versus 4.7
PD-1/PD-L1	Nivolumab	ATTRACTION 2/3rd-line [102]	None/nivolumab versus placebo in Asian patients	Positive (HR 0.63)/5.26 versus 4.14
	Pembrolizumab	KEYNOTE-061/2nd-line [96]	PD-L1/pembrolizumab versus paclitaxel	Negative (HR 0.82)/9.1 versus 8.3
	Pembrolizumab	KEYNOTE-181/2nd-line [97]	PD-L1/pembrolizumab versus chemotherapy	Positive (HR 0.69)/9.3 versus 6.7 in PD-L1 ≥ 10%
	Pembrolizumab	KEYNOTE-062/1st-line [98]	PD-L1 & HER2(−)/pembrolizumab alone or pembrolizumab plus chemotherapy versus chemotherapy alone	Noninferior for Pembrolizumab (HR 0.91)/10.6 versus 11.1. Negative for Pembrolizumab and chemotherapy (HR 0.85)/12.5 versus 11.1
	Avelumab	JAVELIN Gastric 300/3rd-line [103]	None/avelumab versus chemotherapy	Negative (HR 1.1)/4.6 versus 5.0
DNA	TAS-102	TAGS/3rd-line [106]	None/TAS-102 versus placebo	Positive (HR 0.69)/5.7 versus 3.6
EGFR	Cetuximab	EXPAND/1st-line [120]	None/chemotherapy with or without cetuximab	Negative (HR 1.03)/9.4 versus 10.7
	Panitumumab	REAL3/1st-line [121]	None/chemotherapy with or without panitumumab	Negative (HR 1.37)/8.8 versus 11.3
STAT3	Napabucasin	BRIGHTER/2nd-line [132]	None/paclitaxel with napabucasin or placebo	Negative (HR 1.01)/6.93 versus 7.36
PARP	Olaparib	GOLD/2nd-line [137]	None/paclitaxel with olaparib or placebo	Negative (HR 0.79 [97.5% CI 0.63–1.00])/8.8 versus 6.9
MMP-9	Andecaliximab	GAMMA-1/1st-line [138]	HER2(−)/mFOLFOX6 with andecaliximab or placebo	Negative (HR 0.93)/12.5 versus 11.8
MET	Onartuzumab	METGastric/1st-line) [144]	MET and HER2(−)/mFOLFOX6 with onartuzumab or placebo	Negative (HR 0.82)/11.0 versus 11.3
	Rilotumumab	RILOMET-1/1st-line) [143]	MET and HER2(−)/chemotherapy with rilotumumab or placebo	Negative (HR 1.34)/8.8 versus 10.7
mTOR	Everolimus	GRANITE-1/3rd-line) [140]	None/everolimus versus placebo	Negative (HR 0.90)/5.4 versus 4.3

*HER2* human epidermal growth factor receptor 2, *HR* hazard ratio, *PD-1* programmed death-1, *PD-L1* programmed death ligand 1, *DNA* deoxyribonucleic acid, *EGFR* epidermal growth factor receptor, *STAT3* signal transducer and activator of transcription 3, *PARP* poly (ADP-ribose) polymerase, *MMP-9* matrix metalloproteinase 9, *mFOLFOX6* 5-fluorouracil/leucovorin/oxaliplatin, *MET* mesenchymal–epithelial transition, *mTOR* mammalian target of rapamycin

**Table 2:** Completed randomized phase II studies with targeted agents in advanced gastric cancer

Target	Agent	Trial	Selection biomarker/trial design	Progression-free survival benefit/months (experimental versus control)
HER2	Trastuzumab	WJOG7112G (T-ACT)/2nd-line [18]	HER2/paclitaxel with or without trastuzumab	Negative (HR 0.91)/3.68 versus 3.19
Angiogenesis	Sorafenib	STARGATE/1st-line [80]	None/chemotherapy with or without sorafenib	Negative (HR 0.92)/5.6 versus 5.3
	Ziv-aflibercept	MEGA/1st-line [79]	None/mFOLFOX6 with ziv-aflibercept or placebo	Negative (HR 1.11)/9.7 versus 7.4
	Sunitinib	NCT01238055/2nd-line [83]	None/docetaxel with or without sunitinib	Negative (HR 0.77 [95% CI 0.52–1.16])/3.9 versus 2.6 (time to progression)
	Regorafenib	INTEGRATE/3rd-line [81]	None/regorafenib versus placebo	Positive (HR 0.40)/2.6 versus 0.9
	Ramucirumab	RAINSTORM/1st-line [69]	HER2(−)/chemotherapy with ramucirumab or placebo in Asian patients	Negative (HR 1.07)/6.34 versus 6.74
	Ramucirumab	NCT01246960/1st-line [65]	None/mFOLFOX6 with ramucirumab or placebo	Negative (HR 0.98)/6.4 versus 6.7
	Pazopanib	NCT01503372/1st-line [85]	HER2(−)/chemotherapy with or without pazopanib	Negative (HR 0.93)/5.1 versus 3.9
FGFR	AZD4547	SHINE/2nd-line [112]	FGFR/AZD4547 versus paclitaxel	Negative (HR 1.57)/1.8 versus 3.5
Claudin 18.2	IMAB362	FAST/1st-line [118]	Claudin 18.2 & HER2(−)/chemotherapy with or without IMAB362	Positive (HR 0.44)/7.5 versus 5.3
Smoothened (Hedgehog signaling)	Vismodegib	NCT00982592/1st-line [129]	None/mFOLFOX6 with vismodegib or placebo	Negative/7.3 versus 8.0

*HER2* human epidermal growth factor receptor 2, *HR* hazard ratio, *mFOLFOX6* 5-fluorouracil/leucovorin/oxaliplatin, *FGFR* fibroblast growth factor receptor

**Table 3:** Ongoing phase III trials with targeted agents in advanced gastric cancer

Targeted pathway	Agent/selection biomarker	Trial design	ClinicalTrials.gov identifier
HER	Varlitinib/HER1 and HER2	Phase II/III 1st-line comparing varlitinib plus mFOLFOX6 versus placebo plus mFOLFOX6	NCT03130790
	Nimotuzumab/HER1	ENRICH 2nd-line comparing irinotecan with or without nimotuzumab in Asian patients	NCT01813253
Angiogenesis	Ramucirumab/HER2(−)	ARMANI comparing maintenance therapy with ramucirumab plus paclitaxel versus continuation of 1st-line chemotherapy	NCT02934464
	Regorafenib/none	INTEGRATEII 3rd-line comparing regorafenib versus placebo	NCT02773524
	Apatinib/none	ANGEL 3rd-line comparing apatinib versus placebo	NCT03042611
	Fruquintinib/none	FRUTIGA 2nd-line comparing fruquintinib plus paclitaxel versus placebo plus paclitaxel	NCT03223376
	Anlotinib/none	ALTER0503 3rd-line comparing anlotinib versus placebo	NCT02461407
PD-1/PD-L1	Nivolumab with or without ipilimumab/HER2(−)	CheckMate649 1st-line comparing nivolumab plus ipilimumab or nivolumab plus chemotherapy against chemotherapy alone	NCT02872116
	Nivolumab/HER2(−)	ATTRACTION-4 1st-line comparing nivolumab plus chemotherapy versus placebo plus chemotherapy	NCT02746796
	Pembrolizumab/PD-L1	KEYNOTE-063 2nd-line comparing pembrolizumab versus paclitaxel in Asian patients	NCT03019588
	Pembrolizumab/HER2	KEYNOTE-811 1st-line comparing pembrolizumab plus trastuzumab in combination with chemotherapy versus placebo plus trastuzumab in combination with chemotherapy	NCT03615326
	Pembrolizumab/none	KEYNOTE-590 1st-line in esophageal including gastroesophageal junction cancer comparing chemotherapy versus chemotherapy plus pembrolizumab in Chinese patients	NCT03189719
	Avelumab/HER2(−)	JAVELIN Gastric 100 comparing maintenance therapy with avelumab versus continuation of 1st-line chemotherapy	NCT02625610
FGFR2	FPA144/FGFR2 & HER2(−)	FIGHT/1st-line comparing FPA144 plus mFOLFOX6 versus placebo plus mFOLFOX6	NCT03694522
Claudin 18.2	IMAB362/claudin 18.2 & HER2(−)	SPOTLIGHT/1st-line comparing IMAB362 plus mFOLFOX6 versus placebo plus mFOLFOX6	NCT03504397

*HER* human epidermal growth factor receptor, *mFOLFOX6* 5-fluorouracil/leucovorin/oxaliplatin, *PD-1* programmed death-1, *PD-L1* programmed death ligand 1, *FGFR2* fibroblast growth factor receptor 2

## Anti-HER2 antibodies

HER2 is one of four HER RTK family including epidermal growth factor receptor (EGFR, also known as HER1), HER3, and HER4. HER2 does not bind to specific ligands, and transduces cell growth signaling by heterodimerizing with other HER family members [10]. HER2 overexpression is determined by immunohistochemistry (IHC) and/or fluorescence in situ hybridization (FISH), and is seen in about 20% of GC, which results in poor outcome and more aggressive disease course [11].

### Trastuzumab

Trastuzumab is a HER2 monoclonal antibody which leads to cell cycle arrest at G1 and anticancer activity in HER2 overexpressed GC cells [12]. The phase III ToGA enrolled 594 patients with HER2 overexpressed advanced GC in 24 countries including Europe, Central America, South America, and Asia; patients were assigned in a 1:1 ratio to either trastuzumab in combination with chemotherapy (capecitabine or 5-FU plus cisplatin, n = 298) or chemotherapy alone (n = 296) [13]. HER2 overexpression, determined by IHC 3+ and/or positive FISH, was detected in 22% of patients screened for this study. The investigational group with trastuzumab plus chemotherapy achieved longer OS (13.8 months versus 11.1 months, p = 0.0046), longer progression-free survival (PFS) (6.7 months versus 5.5 months, p = 0.0002), and higher response rate (RR) (47% versus 35%, p = 0.0017) than the control group with chemotherapy alone. Toxicity profiles were similar between these two groups. This study has established a practice-changing paradigm in first-line treatment for advanced GC overexpressing HER2.

Subsequently, the phase IIIb HELOISE trial was conducted to compare 2 dose regimens of trastuzumab combined with chemotherapy as a first-line treatment for patients with HER2-positive advanced GC [14]. This study attempted to answer the issue noted in the ToGA study that about one-third of patients assigned to trastuzumab arm were underdosed, which might have resulted in low trough levels of the drug and worse survival. Treatment arms of HELOISE study included loading dose trastuzumab at 8 mg/kg followed by standard trastuzumab maintenance therapy at 6 mg/kg every 3 weeks or loading dose trastuzumab at 8 mg/kg followed by high-dose trastuzumab maintenance therapy 10 mg/kg every 3 weeks until progression. Results showed that the high-dose regimen was associated with increased trastuzumab levels of concentration but did not result in improved OS. The exploratory analyses indicate patients with higher HER2 levels (IHC 2+ and positive FISH or IHC 3+; about 78% of HER2 overexpressors) had a 4.2-month improvement in median OS with trastuzumab (hazard ratio [HR] 0.65); the incidence of HER2 overexpression was more common in GEJ than in the gastric body, and more common in the intestinal than diffuse histology subtype [15]. Others have examined the correlation of biomarkers in responders and resistant patients in trastuzumab-containing regimens. Pietrantonio et al. used panel testing including EGFR/MET/KRAS/PI3K/PTEN mutations and EGFR/MET/KRAS amplification, and found these genomic alterations were more commonly seen in resistant patients than responders; patients with tumors bearing no candidate genomic alterations had a significantly longer OS (16.1 versus 7.6 months; HR 0.38; 95% confidence interval [CI] 0.09–0.75; p = 0.015) [16]. Takahashi et al. examined serum levels of growth factors involved in HER2 signaling in trastuzumab treated GC patients. Hepatocyte growth factor (HGF) was noted to be significantly lower in responders compared with that in non-responders (p = 0.014). Multivariate analyses revealed elevated level of serum HGF was associated with poor OS outcome compared with low level of HGF (adjusted HR 3.857 [95% CI 1.309–11.361]; p = 0.014) [17].

WJOG7112G (T-ACT) was a randomized phase II study conducted in Japan investigating the benefit of adding trastuzumab to paclitaxel beyond first-line treatment in advanced HER2-positive GC (Clinical trial information: UMIN000009297). This study enrolled 91 patients who progressed after first-line chemotherapy with trastuzumab plus fluoropyrimidine and platinum, and randomized to receive paclitaxel or paclitaxel plus trastuzumab. There was no significant difference in PFS as the primary endpoint [18]. Median PFS was 3.19 (95% CI 2.86–3.48) and 3.68 (95% CI 2.76 to 4.53) months in paclitaxel and paclitaxel plus trastuzumab arms, respectively (HR 0.91 [95% CI 0.67–1.22]; p = 0.33). Median OS was 9.95 months in the paclitaxel arm and 10.2 months in the paclitaxel plus trastuzumab arm (HR 1.23 [95% CI 0.75–1.99]; p = 0.20). Biomarker study of this trial showed that two-thirds of patients lost tumor HER2-positivity after the progression of prior trastuzumab-containing chemotherapy [19]. Detected serum neuregulin-1, a ligand of HER3 and an activator of HER2 heterodimerization, was associated with poorer outcomes in the trastuzumab plus paclitaxel arm.

### Pertuzumab

Pertuzumab, a monoclonal antibody against HER2 at a different binding site from trastuzumab, interferes with HER2 dimerization with other HER family members. Combination of pertuzumab and trastuzumab results in stronger inhibition of HER2 signaling and greater therapeutic efficacy compared to trastuzumab alone in breast cancer [20]. JACOB, a phase III study, compared trastuzumab and platinum-based chemotherapy with or without pertuzumab as first-line treatment in HER2 overexpressed advanced GC [21]. More than 700 patients were enrolled in this study from Jun 2013 to Jan 2016. The median OS was 17.5 months in the investigational arm versus 14.2 months in the control arm (HR 0.84 [95% CI 0.71–1.00]; p = 0.0565). Median PFS was 8.5 months and 7.0 months respectively (HR 0.73 [95% CI 0.62–0.86]). This study failed to demonstrate a statistically significant improvement in OS with the addition of pertuzumab to trastuzumab and platinum-based chemotherapy despite of an observed 3.3-month increase in median OS.

### Margetuximab (also known as MGAH22)

Margetuximab is a monoclonal antibody derived from trastuzumab, with a genetically engineered Fc domain to enhance antibody dependent cell-mediated cytotoxicity (ADCC) [22]. Preclinical evaluation showed that while margetuximab maintains HER2-binding properties and exhibits direct anti-proliferative activity of trastuzumab against HER2-expressing cancer cells, the enhanced Fc domain results in improved anti-tumor activity against HER2-expressing as well as HER2 low-expressing cancer cells compared to trastuzumab. A phase I study of margetuximab demonstrated single-agent activity in several HER2-expressing tumor types refractory to trastuzumab including breast cancer and GC [23]. Ex vivo analyses of patients’ peripheral blood mononuclear cells supported the notion of enhanced ADCC of margetuximab compared with trastuzumab. A phase 1b/2, open label, dose-escalation study of margetuximab in combination with pembrolizumab, a PD-1 inhibitor, in patients with advanced GC expressing HER2 has demonstrated promising activity with acceptable tolerability (NCT02689284). Responses were evaluated in 51 patients, 26 of them with GEJ cancer; RR across all patients in the study was 18%. RR was higher in patients with gastric versus GEJ cancer (32% versus 4%), and median PFS was also higher in patients with gastric versus GEJ cancer (5.5 versus 1.4 months) [24]. Biomarker study on pre-treatment HER2 amplification in plasma circulating tumor DNA by next-generation sequencing and PD-L1 expression on archival tumor tissue by IHC (22C3 pharmDx) showed that both HER2 amplification and PD-L1 positivity predicted RR (24% versus 0% [p = 0.0655] and 36% versus 5% [p = 0.0367], respectively) [25]. The RR was 57% and disease control rate (DCR; the proportion of patients with a best overall response of complete response [CR], partial response [PR], or stable disease [SD]) was 86% in patients whose tumors showed both HER2 amplification and PD-L1 expression.

### Antibody–drug-conjugates (ADC)

Trastuzumab emtansine (T-DM1) is an ADC of trastuzumab linked to a mitotic inhibitor emtansine [26]. Binding of T-DM1 to HER2 overexpressing breast cancer cells leads to internalization of the HER2-T-DM1 complex into the cell through receptor-mediated endocytosis, mitotic arrest and apoptosis. In patients with HER2-positive metastatic breast cancer who progressed after lines of trastuzumab-containing therapy, two phase III studies showed significantly improved OS with T-DM1 versus other chemotherapy treatments including lapatinib and capecitabine [27, 28]. The phase II/III GATSBY study compared TDM1 with taxane as second-line treatment in patients with HER2 overexpressing advanced GC. This study enrolled 315 patients who progressed during or after first-line fluoropyrimidine plus platinum therapy with or without HER-2-targeted therapy with OS as the primary endpoint; 228 patients were randomized to weekly T-DM1 and 117 to taxane. 77.4% had previously received HER-2-targeted therapy, and 46.1% were Asian. The median OS was 7.9 months in the T-DM1 versus 8.6 months in the taxane group (HR 1.15 [95% CI 0.87–1.51]). Median PFS was 2.7 months in the T-DM1 group versus 2.9 months in the taxane group [29]. This study failed to demonstrate the benefit T-DM1 as 2nd-line treatment in patients with HER2 overexpressing advanced GC after progressing through trastuzumab-based treatment.

Trastuzumab deruxtecan (DS-8201a) is a HER2-targeted ADC with a humanized HER2 antibody linked to a derivative of the camptothecin analog exatecan (DXd; DX-8951 derivative), a DNA topoisomerase I inhibitor [30]. In preclinical studies, DS-8201a demonstrated efficacy against T-DM1-resistant HER2-expressing as well as low HER2-expressing cancer cells. In a phase I study, DS-8201a demonstrated promising antitumor activity in heavily pretreated patients with HER2 expressing GC with RR of about 40% and acceptable toxicities [31, 32]. A phase II DESTINY-Gastric01 study of DS-8201a is currently underway in patients with HER2-positive advanced GC refractory to trastuzumab (NCT03329690). This study is enrolling patients from Japan and South Korea who have progressed on two prior regimens including fluoropyrimidine agent, platinum agent and trastuzumab [33]. Patients will be randomized 2:1 to DS-8201a alone or physician’s choice of chemotherapy with either paclitaxel or irinotecan. Additionally, this study plans to enroll two non-randomized exploratory cohorts with HER2 low-expressing advanced GC. The primary endpoint of this study is RR, and the secondary endpoints are OS, PFS, safety, etc.

### Bispecific antibodies

ZW25 is a bispecific antibody simultaneously binding two non-overlapping epitopes (ECD2: pertuzumab binding domain, and ECD4: trastuzumab binding domain) of HER2 [34]. Preclinical studies have shown ZW25 enhances HER2 signal blockade and anti-tumor activity in HER2 expressing xenograft mouse models including GC [35]. In a phase I study evaluating ZW25 safety and efficacy in patients with advanced solid tumors expressing HER2, there were 9 response-evaluable GC patients who had progressed from prior trastuzumab containing chemotherapy (NCT02892123). PR was seen in 4 patients (44%), and SD seen in 1 patient (12%) Treatment was well tolerated with most common adverse events (AEs) being diarrhea and infusion reactions, all grade 1 or 2 [36]. These data suggest ZW25 may overcome trastuzumab resistance in GC.

Other HER2 targeted bispecific antibodies undergoing clinical investigation include ertumaxomab targeting HER2 and CD3 on T cells, MM-111 targeting the HER2/HER3 heterodimer and blocking the binding of heregulin and HER3, and activated T cell armed with HER2-targeted bispecific antibody (HER2Bi-aATC) exhibiting significant inhibition in drug-resistant solid tumors [37]. SBT6050 contains Toll-like receptor 8 agonist conjugated to a HER2-directed monoclonal antibody [38]. In preclinical studies, SBT6050 selectively activated innate and adaptive anti-tumor responses by monocytes and macrophages while sparing systemic immune toxicities. This agent is expected to launch first-in-human trials in 2020.

## HER tyrosine kinase inhibitors (TKIs)

### Lapatinib (also known as GW-572016)

Lapatinib is an oral small-molecule TKI that targets both epidermal growth factor receptor (EGFR; HER1) and HER2 [39]. It has shown activity in preclinical studies in HER2-amplified GC [40]. The randomized phase III TRIO-013/Logic trial showed no significant improvement in OS with the addition of lapatinib to capecitabine and oxaliplatin (CapeOx) versus CapeOx as first-line treatment of advanced HER2-overexpressed GC [41]. This study was conducted in 22 countries including North America, South America, Europe, and Asia. It randomized 545 patients in a 1:1 ratio to CapeOx plus lapatinib (n = 272) or CapeOx alone (n = 273). No prior palliative chemotherapy was allowed and no prior adjuvant or neoadjuvant therapy was allowed within the previous 12 months prior to study entry. Initially, the primary endpoint was PFS, however, after the results of the ToGA trial demonstrated an OS benefit with trastuzumab, the primary endpoint of the LOGIC trial was changed to OS with secondary endpoints including PFS and RR. The results demonstrated an OS of 12.2 months in the lapatinib arm versus 10.5 months in the placebo arm, which was not statistically significant (HR 0.91 [95% CI 0.73–1.12]; p = 0.3492). PFS was 6 months in the lapatinib arm versus 5.4 months in the placebo arm (HR 0.82 [95% CI 0.67–1]; p = 0.0381). A significant increase in RR was noted in the lapatinib arm versus the placebo arm (53% versus 39% respectively, p = 0.0031). Subgroup analysis revealed median OS was improved for Asian patients in the lapatinib versus the placebo group (16.5 months versus 10.9 months; HR 0.68 [95% CI 0.48–0.96]; p = 0.0261). In the subgroup excluding Asian patients, there was no significant difference in median OS in the lapatinib (10 months) versus the placebo (9.1 months) arm (HR 1.04 [95% CI 0.79–1.37]; p = 0.7781). Correlative study of HER2 expression in the subjects receiving lapatinib indicated Asian patients and age younger than 60 had a significant improvement in PFS, particularly among those whose cancers exhibited more than fivefold amplification of HER2 [42]. Lapatinib has also been studied in the second line setting in the phase III TyTAN trial, which showed no improved OS when lapatinib was added to paclitaxel compared to paclitaxel alone in HER2 overexpressed advanced GC [43]. Since the TyTAN study was conducted only in Asia, the improved clinical outcome in lapatinib-treated Asian patients noted in the LOGIC study is not confirmed.

### Afatinib

Afatinib is an irreversible pan-HER TKI that has been approved worldwide as a first-line treatment for advanced non-small cell lung cancer that harbors activating EGFR mutations [44]. In vitro study has shown the effects of afatinib on GC cells are independent of activated HER2 but is attenuated by MET amplification [45]. In a phase II study using afatinib alone in trastuzumab-refractory HER2-expressing GC, a DCR of 42% at 4 months was reported [46]. Afatinib is currently being evaluated in several phase II studies in combination with chemotherapy in advanced GC at 1st and 2nd lines (NCT01743365, NCT02501603 and NCT01522768).

### Dacomitinib (also known as PF-00299804)

Dacomitinib is an irreversible pan-HER TKI [47]. In preclinical studies, dacomitinib showed strong activity in HER2-expressing GC cell lines by inhibition of HER family heterodimer formation and enhanced antitumor efficacy of chemotherapeutic and/or molecular-targeted agents including trastuzumab [48]. A phase II study of 27 heavily pretreated patients with advanced HER2-expressing GC from South Korea has demonstrated single-agent activity for dacomitinib with DCR of 40.7% and RR of 7.4% [49]. In this study, higher serum levels of HER2 extracellular domain and lower levels of soluble E-cadherin correlated with increased efficacy of dacomitinib.

### Varlitinib (also known as ARRY-334543)

Varlitinib is a reversible pan-HER TKI [50]. Preclinical studies demonstrated varlitinib exhibits potent antitumor efficacy in patient-derived HER-expressing GC xenograft models [51]. Kim et al. reported the biological activity of varlitinib in HER-expressing advanced GC in a phase IIA study at 2014 European Society for Medical Oncology annual congress; they found suppressed signaling events of HER pathway in tumor samples after treatment with varlitinib. A phase II/III study comparing varlitinib with 5-FU/leucovorin/oxaliplatin (FOLFOX) versus FOLFOX and placebo as first-line treatment in HER1/HER2 co-expressing advanced GC is currently underway (NCT03130790; Table 3).

### Neratinib (also known as HKI-272)

Neratinib is an irreversible pan-HER TKI [52]. A phase III study in patients with early-stage HER2-expressing breast cancer who have finished at least 1 year of post-surgery trastuzumab therapy demonstrated the survival benefit of neratinib versus placebo which led to the approval of U.S. Food and Drug Administration (FDA) in July 2017 [53]. The efficacy of neratinib has been explored in other solid tumors with HER mutations in a phase II basket study (NCT 01953926). Somatic mutations in HER2 may result in constitutive HER2 activation; however this study showed no response from neratinib in 5 GC patients with HER2 mutation [54].

## Angiogenesis targeted agents

Preclinical studies have shown that overexpression of VEGF could induce angiogenesis, and that inhibition of VEGF or VEGF receptor-2 (VEGFR-2) suppresses tumor growth [55]. In GC patients, circulating and tumor concentrations of VEGF are associated with increased tumor aggressiveness and reduced survival [56]. In patients who underwent gastrectomy for GC, those with VEGF-expressing tumors had a significantly worse outcome than those with VEGF-nonexpressing tumors [57].

### Ramucirumab (also known as IMC-1121B)

Ramucirumab is a fully humanized IgG1 monoclonal antibody that binds VEGFR-2 leading to inhibition of receptor-mediated downstream signaling events including ligand-induced proliferation and migration of endothelial cells [58]. The high specificity of ramucirumab and its complete blockade of the VEGFR-2, is believed to provide stronger inhibition of angiogenesis, compared to other angiogenesis inhibitors such as bevacizumab, as well as less off-target effects than TKI [59]. In 2014, ramucirumab was approved by U.S. FDA and European Medicines Agency in advanced GC progressing after prior treatment with fluoropyrimidine or platinum-containing chemotherapy due to two positive phase III trials: the REGARD trial which studied the single-agent efficacy of ramucirumab [60], and the RAINBOW trial which studied the efficacy of ramucirumab in combination with paclitaxel [61].

REGARD randomized 355 patients globally in a 2:1 ratio to either best supportive care in combination with ramucirumab (n = 238) or in combination with placebo (n = 117) with the primary endpoint of OS. This study showed a significantly improved OS with ramucirumab compared to placebo (5.2 months versus 3.8 months; HR 0.776 [95% CI 0.603–0.998]; p = 0.047), which corresponded to a 22% reduction in the risk of death. The survival rates were similar between Asian and non-Asian patients. Secondary endpoints were also significantly improved with ramucirumab, including PFS (2.1 months versus 1.3 months; HR 0.483 [95% CI 0.376–0.620]; p < 0.0001). Biomarker analyses were performed in 152 out of 355 (43%) patients in this study examining the expression of VEGFR-2 and HER2 in tumor samples as well as serum levels of VEGF [62]. None of the biomarkers tested were significantly associated with ramucirumab efficacy; the benefit associated with ramucirumab did not appear to differ by HER2 or VEGFR-2 expression levels in tumor tissues.

RAINBOW randomized 665 patients globally in a 1:1 ratio to ramucirumab 8 mg/kg IV (n = 330) or placebo (n = 335) on days 1 and 15 in combination with paclitaxel 80 mg/m2 IV on days 1, 8, 15 every 28 days. The majority of the patients had received prior fluoropyrimidine combination therapy without anthracycline (75%), while the remainder received prior platinum and fluoropyrimidine combination therapy with anthracycline. The primary endpoint was OS and secondary endpoints included PFS and RR. OS was significantly longer with ramucirumab plus paclitaxel compared to placebo plus paclitaxel (9.6 months versus 7.4 months; HR 0.807 [95% CI 0.678–0.962]; p = 0.017). Patients from Asian countries had a longer median OS independent of treatment arms. Subgroup analyses showed that patients from non-Asian countries benefited more in OS from the addition of ramucirumab to paclitaxel (8.5 months versus 5.9 months for placebo; HR 0.73 [95% CI 0.59–0.91]) than patients from Asian countries, who saw no benefit with the addition of ramucirumab to paclitaxel (12.1 months versus 10.5 months for placebo; HR 0.97 [95% CI 0.73–1.34]). Secondary endpoints such as RR and median PFS were also significantly longer with ramucirumab plus paclitaxel compared to placebo plus paclitaxel; 28% versus 16% (p = 0.0001) for RR, and 4.4 months versus 2.9 months for PFS (HR 0.635 [95% CI 0.536–0.752]; p < 0.0001). Further subgroup analysis of 223 East Asian patients in the RAINBOW study showed the improvements in PFS and RR from ramucirumab in East Asian patients were consistent with the improvements in non-East Asian patients [63]. Drug exposure–response study in East Asian patients receiving ramucirumab showed higher ramucirumab minimum trough concentration at steady state correlated with longer OS and PFS as well as higher incidence of severe neutropenia toxicity [64]. The lack of OS benefit from ramucirumab in East Asian patients might be due to the more frequent use of post-study treatment such as 3rd and 4th line of chemotherapy by East Asian patients (67% in both arms combined) compared to non-East Asian patients (37% in both arms combined).

The combination of ramucirumab and chemotherapy as front-line treatment in advanced GC has been studied in several randomized studies. Yoon et al. reported a phase II trial enrolling patients in USA examining ramucirumab as first-line therapy in patients with advanced GC (NCT01246960) [65]. This study randomized 164 patients to receive FOLFOX with ramucirumab at 8 mg/kg every 14 days or placebo. Compared to placebo, ramucirumab did not improve PFS (6.4 versus 6.7 months; HR 0.98) or OS (11.7 versus 11.5 months; HR 1.08). RAINFALL (NCT02314117), a phase III study, randomized 645 patients with HER2-nonexpressing advanced GC to receive cisplatin plus a fluoropyrimidine with ramucirumab (326 patients) or placebo (319 patients) as first-line treatment [66]. Ramucirumab was given at a higher dose of 8 mg/kg on days 1 and 8 every 21 days in RAINFALL rather than 8 mg/kg every 14 days as in REGARD and RAINBOW trials, since a higher concentration of ramucirumab was shown to correlate with improved OS in both REGARD and RAINBOW [67]. PFS was statistically improved in patients treated with ramucirumab versus placebo (5.7 versus 5.4 months; HR 0.75 [95% CI 0.61–0.94]; p = 0.011), meeting the primary endpoint but with only 9 days of difference. Additionally, there was no OS benefit for patients treated with ramucirumab (11.2 versus 10.7 months; HR 0.96 [95% CI 0.80–1.16]; p = 0.68). RAINSTORM was a randomized phase II study in Japan, South Korea, and Taiwan that evaluated the effectiveness of S-1 and oxaliplatin with ramucirumab (n = 96) or placebo (n = 93) as first-line treatment followed by paclitaxel with ramucirumab as a second-line therapy in patients with HER2-nonexpressing advanced GC (NCT02539225) [68]. Ramucirumab was given at the same dose intensity as that given in RAINFALL for the first-line treatment portion of the study then it was decreased to the same dose intensity as that given in RAINBOW in the 2nd-line portion of the study. There was no significant difference in PFS as the primary endpoint with median PFS of 6.34 months in experimental arm versus 6.74 months in control arm (HR 1.07; p = 0.698) [69].

Overall, these studies demonstrate the activity of ramucirumab in advanced GC leading to regulatory approval of second-line use as a single agent or in combination with paclitaxel without predictive biomarker. There was no OS benefit from the addition of ramucirumab to paclitaxel in East Asian patients seen in the RAINBOW trial, likely due to higher rates of post-study treatment with 3rd- and 4th-line chemotherapy in this cohort. With the negative results from 1st-line studies including RAINFALL and RAINSTORM giving ramucirumab at a higher dose of 8 mg/kg on days 1 and 8 of every 21 days in combination with chemotherapy, ramucirumab is not recommended to be use in the first-line setting. Ramucirumab is currently being investigated as 3rd-line treatment in combination with irinotecan versus irinotecan alone (1:1 randomization) in Japan (RINDBeRG; Clinical trial information: UMIN000023065). This study plans to enroll 400 patients with advanced GC who progress after two or more lines of chemotherapy with platinum, fluoropyrimidines, taxanes, and ramucirumab with primary endpoint being OS [70].

### Apatinib

Apatinib mesylate, formerly known as YN968D1, is a small-molecule TKI that selectively targets VEGFR-2 and binds to its ATP intracellular binding sites leading to the inhibition of phosphorylation and subsequent downstream signaling pathways, including the RAF/MEK/ERK pathway. The result is a decrease in VEGF mediated endothelial cell migration, proliferation, and tumor microvascular density. Apatinib has also been shown to have inhibitory effects on Ret, c-kit, and c-SRC. It has a structure similar to that of vatalinib and is 10 times more potent than vatalinib or sorafenib. Treating Apatinib with several enzymes, such as CYP2C9, CYP2D6, CYP2E2A, and UGT2B7, resulted in the production of four metabolites, with cis-3-hydroxy-apatinib-*O*-glucoronide (M9-2) identified as the major metabolite. Apatinib has a bioavailability of 10–20% after oral intake and is excreted mainly via the feces unmetabolized (69.8%) and via the urine almost completely metabolized (7%). In vivo, apatinib has shown activity in nude mice on various tumors, including GC [71].

Based on the promising results of a phase II trial in advanced GC [72], the apatinib 850 mg once daily dose was chosen for a phase III trial in advanced GC undertaken in 32 centers in China [73]. This was a multi-center, randomized, double-blind, placebo-controlled study that aimed to study the efficacy and safety of apatinib in the third line setting. It randomized 273 patients in a 2:1 ratio to apatinib 850 mg (n = 176) once daily or matching placebo (n = 91) every 28 days. Patients must have progressed on 2 prior lines of therapy to be eligible. Co-primary endpoints were OS and PFS. With regard to patient demographics and baseline characteristics, the percentage of patients with a ECOG performance status of 0 in the apatinib arm was higher than in the placebo arm (27.3% versus 16.5%), but this was not found to be statistically significant (p = 0.0674). The results showed a longer median OS for patients on apatinib compared to placebo (6.5 months versus 4.7 months; HR 0.71 [95% CI 0.54–0.94]; p = 0.149), which translated to a 30% reduction in the risk of death. Median PFS was also improved with apatinib (2.6 months versus 1.8 months for placebo; HR 0.44; 95% CI 0.33–0.61; p < 0.001). Results from the per-protocol population further supported these results with a median OS of 7.6 months with apatinib and 5 months for placebo (HR 0.616 [95% CI 0.447–0.849]; p = 0.0027) and PFS of 2.8 months for apatinib and 1.9 months for placebo (HR 0.455 [95% CI 0.32–0.624]; p < 0.001). In terms of safety, most of the side effects were similar to those reported in previous studies with apatinib as well as with other angiogenesis inhibitors. Some of the most common grade 3 or 4 AEs were hypertension, hand-foot syndrome, proteinuria, fatigue, elevated aminotransferase, and anorexia. There was no significant difference in quality of life measures between the two groups. Pooled analysis of apatinib trials in GC showed that the presence of AEs such as hypertension and hand-foot syndrome in the first 4 weeks was associated with prolonged median OS (169 versus 103 days, log-rank p = 0.0039), and increased disease control rate (54.67 versus 32.77%; adjusted odds ratio 2.67, p < 0.001) [74]. Apatinib is an active agent in GC and is an option for patients who have received two or more lines of therapy. Apatinib has been approved by the China FDA in October 2014 for patients with advanced GC. The U.S. FDA granted orphan-drug designation to apatinib for the treatment of advanced GC in June 2017. Apatinib is currently being investigated in a global phase III clinical trial, which is expected to enroll about 459 patients to evaluate the efficacy of apatinib plus best supportive care versus placebo with best supportive care in patients with advanced GC who have failed two or three lines of therapy (NCT03042611).

### Bevacizumab

Bevacizumab is a monoclonal antibody targeting VEGF-A, a key mediator of angiogenesis. A phase III double-blind and placebo-controlled study (AVAGAST) compared fluoropyrimidine and cisplatin with bevacizumab or placebo as the first-line treatment in 774 patients with advanced GC [75]. This study showed no significant difference in OS (primary endpoint), despite significant improvement in median PFS and RR with the addition of bevacizumab. The subgroup analysis showed improved OS in Pan-American compare to Asian and European patients (11.5 months versus 6.8 months; HR 0.63 [95% CI 0.43 to 0.94]) [76]. Biomarker analysis showed that high baseline plasma VEGF-A levels and low baseline tumor neuropilin-1 expression were associated with improved survival in AVAGAST study [77]. AVATAR, a phase III study of similar design with AVAGAST, enrolled 202 Chinese patients [78]. Similar to AVAGAST, AVATAR did not reach its primary endpoint and showed similar median PFS and RR between the investigational and control arms.

### Ziv-aflibercept

Ziv-aflibercept binds to VEGF-A, VEGF-B and placental growth factor. MEGA was a randomized phase II study comparing FOLFOX with ziv-aflibercept or placebo as 1st-line treatment in advanced GC. This study enrolled 64 patients and showed no difference in the 6-month PFS (60.5% in ziv-aflibercept group versus 57.1% in placebo group; p = 0.080), which was the primary endpoint of the study. The median PFS was 9.7 months in the ziv-aflibercept arm and 7.4 months in the placebo arm (HR 1.11 [95% CI 0.64–1.91]; p = 0.72) [79].

### Sorafenib

Sorafenib is a multi-target small-molecule TKI with inhibitory effect on VEGFR-1, -2, and -3, PDGFR-β, and others. STARGATE, a phase II open-label study, investigated sorafenib plus capecitabine and cisplatin versus capecitabine and cisplatin as the first-line treatment in 195 patients from Korea, China, and Taiwan with advance GC [80]. This study did not demonstrate a significant difference in PFS which was the primary endpoint.

### Regorafenib

Regorafenib is a multi-target small-molecule TKI that targets RTK involved in angiogenesis such as VEGFR-2, and tumor microenvironment (PDGFR-B, FGFR-1). INTEGRATE was a phase II randomized, double-blind, placebo-controlled study conducted to evaluate the efficacy and safety of regorafenib in advanced esophagogastric cancer patients in the second or third line setting and to determine whether regorafenib warrants further evaluation in a phase III study for this indication [81]. It randomized 152 patients in a 2:1 ratio to best supportive care in combination with regorafenib 160 mg orally daily on days 1–21 or in combination with placebo on days 1–21 every 28 days with the primary endpoint of PFS. Baseline demographics were well balanced between both groups and included patients from Australia, New Zealand, Canada, and parts of Asia. The results demonstrated significant improvement in median PFS for regorafenib versus placebo (2.6 months versus 0.9 months for placebo; HR 0.40 [95% CI 0.28 to 0.59]; p < 0.001). Regional differences were found with greater effect in South Korea than in Australia, New Zealand, and Canada combined (HR 0.12 versus 0.61; p < 0.001); but regorafenib was effective in both regional groups. A randomized phase III double-blind placebo-controlled study known as INTEGRATEII is currently underway with the primary endpoint being OS (NCT02773524; Table 3).

### Sunitinib

Sunitinib, a multi-targeted TKI of VEGFR, PDGFR, etc., is approved for the treatment of advanced renal cell carcinoma, pancreatic neuroendocrine tumor, and gastrointestinal stromal tumor. Sunitinib demonstrated promising activity as second-line treatment in advanced GC with OS of 6.8 months and PFS of 2.3 months [82]. A randomized open-label phase II study (NCT01238055) with a primary endpoint of time to progression compared docetaxel with or without sunitinib in the second-line setting following failure of treatment with a fluoropyrimidine and platinum combination for advanced GC [83]. There were 107 patients enrolled from South Korea. The study did not meet its primary endpoint; there was no significant difference in time to progression between docetaxel plus sunitinib and docetaxel alone arms (3.9 months versus 2.6 months; HR 0.77 [95% CI 0.52–1.16]; p = 0.206). However, the objective response rate was significantly higher in the docetaxel plus sunitinib arm (41.1% versus 14.3%, p = 0.002).

### Pazopanib

Pazopanib, a TKI blocking VEGFR-1, -2, -3, c-kit, and PDGFR, is approved in advanced soft tissue sarcoma and renal cell cancer. Median OS of 10 months was seen in a phase II first-line study for advanced GC receiving pazopanib, fluoropyrimidine and platinum agent [84]. In a randomized phase II trial of 1st-line chemotherapy of FOLFOX with or without pazopanib (NCT01503372) enrolling 87 patients with advanced GC not expressing HER2, median PFS was 5.1 months and 3.9 months (HR 0.93 [95% CI 0.56–1.54]); median OS was 10.1 months and 7.0 months (HR 0.80 [95% CI 0.44–1.48]), respectively. Pazopanib in combination with FOLFOX showed marginal efficacy in this randomized phase II study regarding PFS [85]. Kim and others conducted a single-arm phase II first-line study with pazopanib plus CapeOx in 66 patients with advanced GC from South Korea; the result showed that the median PFS and OS were 6.5 months (95% CI 5.6–7.4) and 10.5 months (95% CI 8.1–12.9), respectively [84]. IHC analysis of FGFR2 and VEGFR2 was performed in 54 patients in this study, a significant difference in PFS was seen between patients who were positive and negative for FGFR2 expression by IHC (8.5 versus 5.6 months; p = 0.050) [86].

## Inhibitors of PD-1 and PD-L1

PD-1 and its ligands, PD-L1 and PD-L2, negatively regulate T cell activation. In tumor tissues, when PD-1 binds to PD-L1, it inhibits effector T-cell function resulting in the suppression of antitumor immune response and promotion of tumor growth. PD-L1 is constitutively expressed in various tissues and can also be expressed in several tumor types, including GC [87]. PD-L1 expression determined by IHC is found in many solid tumors including about 40% of GC, and can lead to aggressive tumor behavior and poor clinical outcomes [88, 89]. There have been more than five antibodies against PD-1/PD-L1 approved for clinical use as cancer therapeutics either as single agent or in combination with chemotherapy [90].

### Pembrolizumab (also known as MK-3475)

Pembrolizumab is a humanized IgG4-k monoclonal anti-PD1 antibody blocking the interaction between PD-1 and its ligands [87]. Pembrolizumab is approved by US FDA for the treatment of a wide variety of advanced malignancies including melanoma, lung cancer, head and neck cancer, classical Hodgkin lymphoma, primary mediastinal large B-cell lymphoma, urothelial carcinoma, MSI-high cancer, cervical cancer, and hepatocellular carcinoma.

The KEYNOTE-012 (NCT01848834) is a basket phase Ib study of pembrolizumab in patients with advanced solid tumors; in 36 evaluable PD-L1-positive GC; the result showed 22% RR, and 6-month PFS and OS rate of 24% and 69%, respectively [91]. Efficacy of pembrolizumab in patients with MSI-high or mismatch repair deficient solid tumors was demonstrated in two phase II studies: KEYNOTE-164 (colorectal cancer) and KEYNOTE-158 (non-colorectal) [92]. There were 11 GC in KEYNOTE-158. RR was 27.9% in KEYNOTE-164, and 37.7% in KEYNOTE-158; 6-month OS rate was 87% and 73%, respectively. Based on these results, the U.S. FDA approved pembrolizumab in adult patients with unresectable or metastatic solid tumors harboring MSI or DNA mismatch repair deficiency in May 2017.

The phase II KEYNOTE-059 in advanced GC was a 3-cohort study: cohort 1 enrolled 259 patients with at least two prior lines of therapy to pembrolizumab alone; cohort 2 enrolled 25 patients to first-line pembrolizumab and cisplatin plus 5-FU or capecitabine; cohort 3 enrolled 31 patients with PD-L1 positive tumors to first-line pembrolizumab alone. PD-L1 expression was assayed by IHC study using an FDA-approved 22C3 pharmDx kit; PD-L1 positivity was determined to be 1% or higher of combined positive score (CPS) by dividing the number of PD-L1 staining cells including tumor and inflammatory cells over total number of viable tumor cells. In cohort 1, the RR was 15.5% in PD-L1-positive tumors (148 patients) versus 6.4% in PD-L1-negative tumors (109 patients); the duration of response was 16.3 versus 6.9 months, respectively [93]. The RR was 57.1% in 7 patients with MSI-high tumors versus 9% in 167 patients with non-MSI-high tumors. Eighteen-gene T-cell inflamed gene expression profile was investigated in pretreatment tumor samples [94]; improved response was seen in patients with more T-cell-inflamed tumors. Patients in cohort 2 had a median follow-up of 14 months; the RR was 60%; patients with PD-L1–positive tumors had a higher RR than those with PD-L1–negative tumors (73% versus 38%). The RR was 25.8% in cohort 3 with median follow-up of 18 months [95]. Based on the results of cohort 1 in KEYNOTE-059, in September 2017, the U.S. FDA approved pembrolizumab for the treatment of patients with PD-L1–positive recurrent or advanced GC after receiving 2 or more lines of treatment including fluoropyrimidine and platinum agents. This accelerated approval requires a confirmatory trial.

KEYNOTE-061 (NCT02370498) was a phase III study comparing pembrolizumab versus paclitaxel as second-line therapy for advanced GC, whose disease progressed after first-line treatment with platinum and fluoropyrimidine doublet therapy [96]. The primary endpoints were PFS and OS in patients whose tumors expressed PD-L1 (CPS ≥ 1); secondary endpoints included PFS, OS and RR regardless of PD-L1 expression. Among 592 patients enrolled in this study, there were 395 patients with tumors expressing PD-L1 (CPS ≥ 1): 196 assigned to pembrolizumab and 199 patients to paclitaxel. In this cohort, median OS was 9.1 months with pembrolizumab versus 8.3 months with paclitaxel (HR 0.82; one-sided p = 0.042). Median PFS was 1.5 months with pembrolizumab and 4.1 months with paclitaxel (HR 1.27 [95% CI 1.03–1.57]). Pembrolizumab did not improve OS or PFS compared with paclitaxel as second-line therapy. In a post-hoc analysis, patients with tumors expressing higher level of PD-L1 (CPS ≥ 10) seemed to benefit more from pembrolizumab than with paclitaxel (HR 0.64 [95% CI 0.41–1.02]). This finding has been confirmed in the KEYNOTE-181 and KEYNOTE-062 trials.

KEYNOTE-181, a phase III global open-label randomized study, evaluated pembrolizumab versus investigator’s choice of chemotherapy as second-line treatment in 628 patients with advanced or metastatic esophageal cancer including Siewert type I GEJ adenocarcinoma. Two-thirds of the study population had squamous cell carcinoma of the esophagus [97]. Patients were randomized to receive either pembrolizumab or investigator’s choice of paclitaxel, docetaxel, or irinotecan, and stratified by histology (squamous cell carcinoma versus adenocarcinoma) and region (Asia versus rest of the world). The three co-primary endpoints were OS in the intent-to-treat population, the squamous cell carcinoma subgroup (n = 401), and the subgroup with tumors harboring PD-L1 ≥ 10 by CPS (n = 222). The results showed that pembrolizumab did not improve OS or PFS in the overall population, but did improve OS in the subgroup for patients with tumors expressing PD-L1 ≥ 10 by CPS. In the intent-to-treat analysis, median OS was 7.1 months in each arm (HR 0.89; p = 0.0560). In about one-third of the study population with expression of PD-L1 ≥ 10 by CPS in tumors, the median OS was 9.3 months with pembrolizumab versus 6.7 months with chemotherapy (HR 0.69; p = 0.0074), and the 12-month survival rate in pembrolizumab group was 43% versus 20% in patients receiving chemotherapy, respectively. These data support pembrolizumab as a second-line treatment option for advanced esophageal cancers and GC with PD-L1 ≥ 10 by CPS. On July 30, 2019, the US FDA approved pembrolizumab for patients with recurrent, locally advanced or metastatic, squamous cell carcinoma of the esophagus whose tumors express PD-L1 ≥ 10 by CPS, with disease progression after one or more prior lines of systemic therapy.

The phase 3 KEYNOTE-062 (NCT02494583) compared pembrolizumab alone or in combination with chemotherapy versus chemotherapy alone as first-line therapy for PD-L1 expressing and HER2 nonexpressing advanced GC. This study enrolled 763 patients with PD-L1 CPS ≥ 1 and HER2-negative disease, and the results were reported in the 2019 annual meeting of American Society of Clinical Oncology [98]. Single-agent pembrolizumab arm was found to be noninferior to chemotherapy with median OS 10.6 versus 11.1 months (HR 0.91). The greatest benefit of pembrolizumab was seen in patients with CPS ≥ 10, similar to the results seen in the KEYNOTE-181; 2-year OS was 39% versus 22% for chemotherapy, and median OS was 17.4 months versus 10.8 months for chemotherapy. Combining pembrolizumab with chemotherapy did not improve OS compared with chemotherapy alone (HR 0.85, OS 12.5 versus 11.1 months for patients with a CPS ≥ 1).

Pembrolizumab has been shown to overcome clinical resistance to trastuzumab in patients with advanced breast cancer overexpressing HER2 and PD-L1 positive advanced breast cancer. This was demonstrated in a single-arm phase Ib/II PANACEA trial (IBCSG 45-13/BIG 4-13/KEYNOTE-014), which enrolled 58 patients with HER2 expressing advanced breast cancer previously treated with trastuzumab or T-DM1. All patients received pembrolizumab and trastuzumab, and six (15%; 90% CI 7–29) of the 40 PD-L1-positive patients achieved an objective response [99]. Janjigian et al. reported preliminary findings of an ongoing single-arm phase II trial using pembrolizumab with trastuzumab and chemotherapy with CapeOX or FOLFOX as front-line treatment in advanced HER2-positive GC (NCT02954536) [100]. In this study 35 patients received treatment (14 patients with esophageal cancer, 12 with GEJ cancer, and 9 with GC). Of the 32 evaluable patients, 87% experienced a response, including a CR and SD in 9% and 11% of patients, respectively. After 6.6 months’ follow-up (range 0.03–23.3 months), the median progression-free survival was 11.4 months, and 67% of patients were progression-free at 6 months. Median OS was not reached, and at 12 months, 76% of patients were alive. KEYNOTE-811, a phase III randomized study comparing trastuzumab plus chemotherapy with or with pembrolizumab as 1st-line treatment in patients with advanced GC overexpressing HER2, is ongoing (NCT03615326; Table 3).

The double-blind, placebo-controlled, randomized phase III KEYNOTE-590 trial (NCT03189719) is examining cisplatin and 5-FU with pembrolizumab or placebo as first-line treatment for approximately 700 patients with advanced or metastatic esophageal cancer including GEJ. The trial has an estimated completion date of August 2021 (Table 3). KEYNOTE-811 (NCT03615326) is a phase III trial comparing pembrolizumab plus trastuzumab in combination with chemotherapy versus placebo plus trastuzumab in combination with  chemotherapy as first-line treatment in HER2-positive advanced GC; this study plans to enroll 730 patients (Table 3).

### Nivolumab (also known as ONO-4538, BMS-936558 or MDX-1106)

Nivolumab is a human monoclonal IgG4 antibody which blocks the human PD-1 receptor [101]. Phase III study ATTRACTION-2 (ONO-4538-12) evaluated the efficacy and safety of nivolumab as salvage treatment after failure of at least two lines of  standard chemotherapy treatment for advanced GC. This study randomized 493 patients from Japan, South Korea and Taiwan, in a 2:1 ratio to receive nivolumab (N = 330) or placebo (N = 163). The primary endpoint was OS, with 5.32 months in nivolumab versus 4.14 months in placebo group (HR 0.63 [95% CI 0.50–0.78]; p < 0.0001). The 12-month OS rate was 26.6% in nivolumab versus 10.9% in the placebo group [102]. The RR was 11.2% with nivolumab versus 0% with placebo (p < 0.0001); a median duration of response of 9.53 months in nivolumab group was observed. Median PFS was 1.61 months with nivolumab versus 1.45 months with placebo (HR 0.60 [95% CI 0.49–0.75]; p < 0.0001). Tumor samples were available in 40% of all enrolled patients, and PD-L1 expression was assessed by IHC (28-8 pharmDx assay). However, PD-L1 status was not predictive of survival with nivolumab therapy; survival benefit from nivolumab was seen irrespective of PD-L1 expression in the exploratory analysis. In September 2017, Japan Ministry of Health, Labor and Welfare approved nivolumab for the treatment of unresectable advanced or recurrent GC after progressing through chemotherapy based on this study. ATTRACTION-3, a randomized phase 3 trial comparing nivolumab with chemotherapy as second-line treatment in patients with advanced esophageal cancer without the requirement of PD-L1 expression, has shown a statistically significant OS benefit in a press release in January 2019. Preliminary data from the ATTRACTION-4 study have shown RR of 70% when nivolumab is combined with chemotherapy in the first-line treatment of advanced GC (Table 3). The ongoing CheckMate 649 is a randomized phase III study of nivolumab plus ipilimumab or nivolumab plus chemotherapy versus chemotherapy alone in patients with previously untreated advanced GC not expressing HER2. This study plans to enroll more than 1200 patients regardless of PD-L1 status with primary endpoint being OS (Table 3).

### Avelumab

Avelumab, an anti-PD-L1 antibody, blocks the binding of PD-L1 to PD-1. Avelumab has been shown to unleash T cell-mediated antitumor immune response in preclinical study. JAVELIN Gastric 300 (NCT02625623), a phase III international open-label randomized trial, compared avelumab versus physician’s choice of protocol-specified chemotherapy (paclitaxel 80 mg/m^2^ on days 1, 8, and 15 or irinotecan 150 mg/m^2^ on days 1 and 15, each of a 4-week treatment cycle) in 371 patients with advanced GC progressing after two prior chemotherapeutic regimens [103]. The trial failed to meet its primary end point of improving OS (4.6 versus 5.0 months; p = 0.81) or the secondary end points of PFS (1.4 versus 2.7 months; p > 0.99) or RR (2.2% versus 4.3%) in the avelumab versus chemotherapy arms, respectively.

## DNA inhibitor

TAS-102 (Taiho Pharmacuetical, Tokyo, Japan) is an oral nucleoside inhibitor that is a combination of trifluridine, an antineoplastic thymidine-based nucleoside analog, and tipiracil hydrochloride which is a thymidine phosphorylase (TP) inhibitor [104]. Oral trifluridine is rapidly degraded to its inactive form by TP in the intestines and liver. Tipiracil selectively inhibits TP in human liver and intestines, which increases the bioavailability and maximum plasma concentration and antitumor activity of oral trifluridine, thus allowing for the clinically feasible oral administration of TAS-102. The primary anticancer mechanism of TAS-102 is distinct from 5-FU in that through incorporation into DNA, trifluridine induces DNA dysfunction such as DNA strand breaks. TAS-102 is approved by US FDA for metastatic colorectal cancer. Bando et al. reported a phase II trial EPOC 1201 which enrolled 29 Japanese patients with refractory advanced GC [105]. All patients had received one or two prior chemotherapy treatments containing fluoropyrimidine, platinum agents, and taxanes or irinotecan. TAS-102 was given at a dose of 35 mg/m^2^ orally twice daily on days 1–5 and 8–12 of a 28-day cycle. The primary endpoint was DCR. At 8 weeks, the DCR was 67.9%, median PFS was 2.9 months, and median OS was 8.7 months. The trial also assessed the pharmacokinetic profile for the 35 mg/m^2^ twice daily dose. There were no differences in the peak serum concentration, area under the plasma-concentration curve, or time of peak serum drug concentration between patients with or without prior gastrectomy. The most common grade 3 and 4 AEs effects were neutropenia, leukopenia, anemia, and anorexia.

TAGS was a phase III randomized, double-blind, placebo-controlled, multicenter trial in 507 patients with advanced GC with ECOG performance status of 0 or 1 who have received at least two previous chemotherapy regimens [106]. Patients were randomized in a 2:1 ratio to TAS-102 plus best supportive care or placebo plus supportive care. The primary endpoint was OS. The primary cancer was gastric in 71% and GEJ in 29% of patients; 63% of patients in each arm had three or greater prior treatments and 44% in each arm had a prior gastrectomy. Median OS was 5.7 months for TAS-102 versus 3.6 months for the placebo group (HR 0.69; p = 0.00029). Compared with placebo, TAS-102 was also associated with significant improvements in PFS, the proportion of patients achieving disease control, and time to deterioration of ECOG performance status. In terms of safety, overall TAS-102 was well tolerated in this study. Serious AEs of any cause were reported in 43% of patients in the TAS-102 group and 42% in the placebo group. The most frequent grade 3 or 4 AEs were consistent with previous studies—neutropenia and anemia in the TAS-102 group versus abdominal pain and general deterioration of physical health in the placebo group. The results of this study demonstrate that TAS-102 could represent a new treatment option for patients with heavily pretreated advanced GC. In February 2019, the US FDA approved TAS-102 in patients with advanced GC previously treated with at least 2 prior lines of chemotherapy that included a fluoropyrimidine, a platinum, either a taxane or irinotecan, and if appropriate, HER2-targeted therapy.

## FGFR inhibitors

FGFR is a transmembrane receptor family with four members (FGFR 1–4) binding to fibroblast growth factors [107]. The signaling of FGFR utilizes mitogen-activated protein kinase and PI3K-AKT pathways. Dysregulation of the FGFR signaling is associated with the development and progression of multiple malignancies [108]. Based on the TCGA dataset, the prevalence of FGFR genomic alterations in GC is 5–10%, and FGFR2b overexpression and FGFR2 gene amplification is associated with a poor prognosis in advanced GC.

### AZD4547

AZD4547 is a TKI targeting FGFR1/2/3, but also inhibits colony stimulating factor 1 receptor, and vascular endothelial growth factor receptor 2 [109]. AZD4547 exerted anti-cancer effect in FGFR amplified GC xenograft models. AZD4547 showed good safety profile without dose-limiting toxicities in a phase I study of patients with advanced malignancies, with SD more than 4 weeks noted in 70% of patients [110]. Common AEs were gastrointestinal toxicities, fatigue, hyperphosphatemia, stomatitis, ocular toxicity, etc. [110, 111]. SHINE, a randomized phase II trial, compared AZD4547 versus paclitaxel in 71 advanced GC patients with FGFR2 amplification as second-line therapy (NCT01457846) [112]. PFS was 1.8 months in AZD4547 versus 3.5 months in paclitaxel arm, and exploratory biomarker analyses revealed significant intratumor heterogeneity of FGFR2 amplification and poor concordance between amplification and FGFR2 mRNA expression. Therefore a suboptimal biomarker selection strategy might have explained why AZD4547 did not significantly improve PFS versus paclitaxel in this cohort of patients with advanced GC.

### FPA144 (aka bemarituzumab)

FPA144, an FGFR2b-specific antibody, prevents the binding of FGFs to FGFR2b, leading to growth inhibition of cancer. A phase 1 study of FPA144 showed no dose-limiting toxicities with DCR of 55.6% and RR of 22% [113]. The FIGHT study (FPA144-004; NCT03343301) is a global, randomized, double-blind, placebo-controlled phase 3 trial evaluating FOLFOX6 with FPA144 as first-line treatment in patients with advanced GC harboring FGFR2b overexpression or FGFR2 amplification. The primary endpoint is OS and secondary endpoints include investigator-assessed PFS and RR. The results from the dose finding phase I lead-in evaluation of the combination showed acceptable safety to proceed with the recommended dose in the phase III portion of the FIGHT study [114].

## Claudin 18.2 inhibitor

Claudin-18 is a tight junction protein, and its splice variant 2 (CLDN18.2) is expressed in about 50–70% of GC [115]. During malignant transformation, the disruption of cell polarity leads to the relocation of CLDN18.2 to GC surface making it targetable by IMAB362 (zolbetuximab), which is a chimeric IgG1 monoclonal antibody [116]. Preclinical evaluation showed IMAB362 mediated antibody-dependent cellular cytotoxicity as well as complement-dependent cytotoxicity against GC cell lines expressing CLDN18.2; improved antitumor activity was noted in xenografted mice treated with IMAB362 plus chemotherapy compared with mice treated with chemotherapy alone [117]. Combination of IMAB362 with chemotherapy using epirubicin, oxaliplatin, and capecitabine showed significantly improved PFS and OS versus chemotherapy alone in FAST study, a randomized phase II first-line study in 161 patients with advanced HER2-negative GC expressing CLDN18.2 (≥ 2+ staining intensity with the anti-CLDN18 43-14A monoclonal antibody in ≥ 40% tumor cells) (NCT01630083). When comparing chemotherapy plus IMAB362 versus chemotherapy alone, the median PFS was 7.5 versus 5.3 months (HR 0.44 [95% CI 0.29–0.67]; p < 0.0005), and the median OS was 13 versus 8.4 months (HR 0.56; p = 0.0008), respectively [118]. The RR was 39% with the combination of IMAB362 plus chemotherapy and 25% with chemotherapy alone; CR rate of 10.4% and 3.6%, respectively. For patients with low versus high CLDN18.2 expression (defined as ≥ 2+ intensity in ≥ 70% tumor cells), the median progression-free survival was 5.6 versus 7.2 months (HR 0.36; p < 0.0005), and median OS was 9 versus 16.7 months (HR 0.45; p < 0.0005), respectively. The combination of IMAB362 and chemotherapy has promising activity in patients with GC expressing CLDN18.2. A phase III global study (SPOTLIGHT; Table 3) of IMAB362 plus FOLFOX versus FOLFOX plus placebo as first-line treatment in patients with CLDN18.2-positive and HER2-negative advanced GC has been launched in 2018 (NCT03504397).

## EGFR targeted agents

EGFR is frequently overexpressed in GC, and this overexpression correlates with poor prognosis [119]. Two phase III studies, EXPAND (cetuximab) and REAL3 (panitumumab) comparing first-line chemotherapy with or without anti-EGFR monoclonal antibody, failed to meet their primary endpoints with PFS in EXPAND and OS in REAL3 [120, 121]. The EXPAND trial enrolled 904 advanced GC patients who were randomized to chemotherapy (capecitabine plus cisplatin) or chemotherapy plus cetuximab [120]. The addition of cetuximab to chemotherapy did not improve PFS (the primary endpoint; 5.6 months for chemotherapy versus 4.4 months for chemotherapy plus cetuximab) or OS (10.7 months for chemotherapy versus 9.4 months for chemotherapy plus cetuximab). The level of EGFR expression determined by IHC did not correlate with treatment response in either treatment group. The REAL3 phase II/III study randomized 553 patients with advanced GC to receive chemotherapy (epirubicin, oxaliplatin, and capecitabine) or reduced-dose chemotherapy plus panitumumab [121]. The addition of panitumumab to chemotherapy showed decreased OS (8.8 months) than chemotherapy alone (11.3 months). Retrospective biomarker study in REAL3 showed mutations either in KRAS (5.7%) or PIK3CA (2.5%) were negative prognostic factors.

Nimotuzumab is a humanized IgG1 monoclonal antibody against EGFR, which has been shown to exhibit anti-tumor effects by regulating antibody-dependent cellular and complement-dependent cytotoxicity, inhibiting proliferation and angiogenesis [122]. Nimotuzumab’s bivalent binding mechanism also allows for selective binding on cells that express moderate to high levels of EGFR expression, which may reduce the incidence of off-target toxicities such as skin rash [123]. It has been approved for the treatment of advanced head and neck cancer in China and other countries except USA [124]. Satoh and others conducted a randomized phase II 2nd-line study in advanced GC comparing nimotuzumab plus irinotecan versus irinotecan alone in 83 patients from Japan and South Korea, which did not demonstrate statistically significant improvement in PFS as primary endpoint [125]. Subgroup analysis of this study showed improved PFS and OS in patients with high EGFR-expression tumors who received nimotuzumab plus irinotecan. The ongoing Phase III ENRICH study plans to randomize 400 patients with advanced GC from Japan, South Korea and Taiwan to receive either nimotuzumab and irinotecan or irinotecan monotherapy as 2nd-line treatment with the primary endpoint as OS (NCT01813253; Table 3).

## Inhibitors of cancer stem cells (CSC)

CSC can generate tumor cells and usually remain quiescent for extended periods of time. CSC exhibit activation of signaling involved in development and tissue homeostasis, including the Notch, Hedgehog, and WNT/β-catenin [126]. CSC are frequently resistant to chemotherapy and radiation treatment, and contribute to drug resistance and treatment failure [127].

### Vismodegib

CD44 is a gastric CSC marker, and the Hedgehog signaling maintains malignant transformation phenotypes in CD44 expressing GC. A preclinical study has shown inhibition of Hedgehog signaling leads to reversal of chemotherapy resistance in CD44 expressing GC cells [128]. Vismodegib is an orally-administrated small-molecule inhibitor of the Hedgehog pathway; vismodegib combined with FOLFOX has been studied in a randomized phase II study as first-line treatment for advanced GC (NCT00982592). This study randomized 124 patients 1:1 to receive either FOLFOX plus vismodegib or FOLFOX plus placebo. The primary endpoint was PFS, and the addition of vismodegib to FOLFOX did not improve PFS [129].

### Napabucasin (aka BBI-608)

Napabucasin is an orally administered signal transducer and activator of transcription 3 (STAT3) inhibitor. It inhibits CSC by blocking phosphorylated STAT3 and downregulating β-catenin [130]. A preclinical study has shown potent synergistic antitumor activity when combined with paclitaxel. In a phase II study of patients with advanced GC, combination of napabucasin and weekly paclitaxel demonstrated encouraging activity in patients who received only 1 prior line of therapy without taxane [131]. Based on this promising result, the US FDA has granted an orphan drug designation to napabucasin in advanced GC. The BRIGHTER phase III study (NCT02178956) randomized 714 patients from North America, South America, Europe, Australia, and Asia with advanced GC following platinum/fluoropyrimidine-based chemotherapy to napabucsin plus paclitaxel versus placebo plus paclitaxel alone as second-line treatment. The results presented by Shah et al. in 2018 annual meeting of American Society of Clinic Oncology (ASCO) showed napabucasin plus paclitaxel did not improved OS or PS as 2nd line treatment in patients with advanced GC [132]. The median OS was 6.93 months versus 7.36 months in napabucasin plus paclitaxel versus placebo plus paclitaxel, respectively (HR 1.01; 95% CI 0.86–1.20; p = 0.8596). The median PFS was 3.55 months versus 3.65 months in napabucasin plus paclitaxel versus placebo plus paclitaxel, respectively (HR 1.00 [95% CI 0.84–1.17]; p = 0.9679).

## Poly (adp-ribose) polymerase (PARP) inhibitor

PARP is a family of nuclear enzymes that regulates the repair of DNA single-strand breaks through the base-excision repair pathway [133]. PARP inhibitors such as olaparib trap inactivated PARP on to single-strand DNA breaks, preventing repair and leading to double-strand DNA breaks [134]. Cancer cells with BRCA1/2 mutation or ataxia telangiectasia mutated (ATM) deficiency cannot repair double-strand DNA breaks and are thus sensitive to PARP inhibition leading to synthetic lethality. Olaparib has been approved by the U.S. FDA in previously treated BRCA-mutated advanced ovarian or breast cancer. Preclinical data showed that GC cells with ATM deficiency were associated with olaparib sensitivity; approximately 10–20% of GC tumor samples exhibit low or undetectable ATM expression by IHC [135]. Bang et al. conducted a randomized phase II study comparing the efficacy of olaparib plus paclitaxel versus paclitaxel as second-line therapy in Asian patients with advanced GC (NCT01063517) [136]. This study was enriched for patients with ATM deficiency (50% randomized versus 14% screening prevalence). There was no difference in PFS (3.9 versus 3.6 months, respectively). There was a statistically significant improvement in OS from olaparib plus paclitaxel regardless of ATM expression (13.1 versus 8.3 months for paclitaxel, respectively; HR 0.56 [80% CI 0.41 to 0.75]; p = 0.005). For patients with ATM deficiency, the median OS was not reached in experimental arm versus 8.2 months in paclitaxel alone arm (HR 0.35 [80% CI 0.22 to 0.56]; p = 0.002). The phase III GOLD study randomized 525 patients who progressed after first-line treatment in China, Japan, South Korea, and Taiwan to receive olaparib plus paclitaxel or placebo plus paclitaxel. 94 patients were determined to have ATM-negative tumors (48 in the olaparib plus paclitaxel group and 46 in the placebo plus paclitaxel group). The study missed the primary endpoint; OS was not significantly improved in the experimental arm of overall patient population (8.8 months versus 6.9 months for placebo; HR 0.79 [97.5% CI 0.63–1.00]; p = 0.026) or in the ATM-negative population (12.0 months versus 10.0 months; HR 0.73 [97.5% CI 0.40–1.34]; p = 0.25) [137].

## Cell adhesion inhibitor

Andecaliximab (aka GS-5745) is a humanized monoclonal antibody that targets matrix metalloproteinase 9 (MMP-9), which is implicated in pro-tumorigenic processes. A phase III study comparing FOLFOX with or without andecaliximab as first-line treatment in patients with HER2-negative advanced GC showed no OS improvement in the investigational arm (GAMMA-1; NCT02545504) [138].

## Inhibitors of mTOR and c-MET/HGF

HGF and its receptor c-MET pathway inhibitors and mTOR inhibitors have been investigated in phase III trials but failed to demonstrate a survival benefit. The mTOR is a protein kinase responsible for regulation of cell growth, proliferation and angiogenesis. Activation of mTOR pathway is associated with worse outcomes in GC [139].

### Everolimus

Everolimus is an oral mTOR inhibitor which demonstrated promising efficacy in a phase II study of advanced GC. Two phase III studies in the second-line setting with either everolimus alone or in combination with paclitaxel showed no improved OS benefit compared to placebo control. The phase III GRANITE-1 study enrolled 656 patients with advanced GC having progressed through first- or second-line treatment; patients were randomized to receive everolimus which is an inhibitor of m-TOR, versus placebo. There was no significant difference in OS; median OS was 5.4 months in the everolimus versus 4.3 months in the placebo group (HR 0.90 [95% CI 0.75 to 1.08]; p = 0.124) [140].

### Rilotumumab and onartuzumab

HGF receptor is encoded by the MET oncogene, and plays an important role in carcinogenesis by activating signaling pathways through RAS, PI3K, and STAT3 [141]. In GC, MET overexpression is associated with a poor prognosis and a more aggressive disease [142]. Two phase III studies using MET inhibitors, either rilotumumab or onartuzumab, in combination with chemotherapy failed to demonstrate improved survival compared to chemotherapy alone.

Rilotumumab is a fully human monoclonal antibody designed to inhibit the MET ligand, HGF. The mechanism of action is designed to selectively bind and neutralize HGF to block its interaction with the MET receptor, ultimately impeding the HGF/MET signaling pathway. RILOMET-1 is a phase III study evaluating rilotumumab plus chemotherapy with epirubicin, cisplatin and capecitabine as first-line therapy in MET-positive and HER2-negative advanced GC. This study randomized 609 patients in 1:1 to chemotherapy plus placebo or chemotherapy plus rilotumumab (NCT01697072). The results showed no survival benefit in the experimental arm. Median OS was 8.8 months in the rilotumumab group compared with 10.7 months in the placebo group (stratified HR 1.34 [95% CI 1.10–1.63]; p = 0.003) [143].

Onartuzumab (formally called MetMAb and PRO 143966) is an anti-MET receptor monoclonal antibody. MetGastric was a randomized phase III study of FOLFOX with or without onartuzumab as first-line treatment in patients with MET-positive and HER2-negative advanced GC. The addition of onartuzumab to FOLFOX did not improve clinical benefits in the overall or MET 2+/3+ populations (NCT01662869) [144].

## Mitogen-activated protein/extracellular signal-regulated kinase (MEK) inhibitor

MEK plays an essential role in RAS/RAF/MEK/extracellular signal-regulated kinase (ERK) cell signaling pathway, which is frequently dysregulated in human cancers. Selumetinib (AZD6244; ARRY-142866) is a TKI of MEK. The correlation between MEK signature, KRAS alteration and treatment response to selumetinib has been demonstrated in GC cell lines [145].

A phase II study of selumetinib plus docetaxel was conducted in South Korea as second-line chemotherapy in advanced GC using molecular screening to identify patients with KRAS mutant, KRAS amplified or wild-type KRAS with MEK signature (ClinicalTrials.gov Identifier: NCT02448290) [146]. Selumetinib was given orally 75 mg twice a day continuously; docetaxel was administered intravenously at 60 mg/m^2^ every 21 days. The primary endpoint was RR, and the secondary endpoints were to perform pre-planned analysis using circulating tumor DNA (ctDNA) and tumors to identify an optimal biomarker for selumetinib. Of 25 patients treated, PR was noted in 7 patients as well as SD in 8 patients, progressive disease in 6 patients, and non-evaluable disease in 4 patients. The RR was 28.0% (95 CI 0.12–0.49). The treatment was well tolerated without grade 3 or 4 AE. The pre-planned biomarker analysis showed that one patient with typical KRAS mutation (at codon 12) and one patient with KRAS amplification plus high MEK signature achieved PR. Three patients with atypical KRAS mutation and low/intermediate MEK signature showed progressive disease. This study has established the efficacy and safety of selumetinib plus docetaxel as 2nd line therapy in advanced GC with certain KRAS genomic alterations.

## Conclusions

**Fig. 1:**
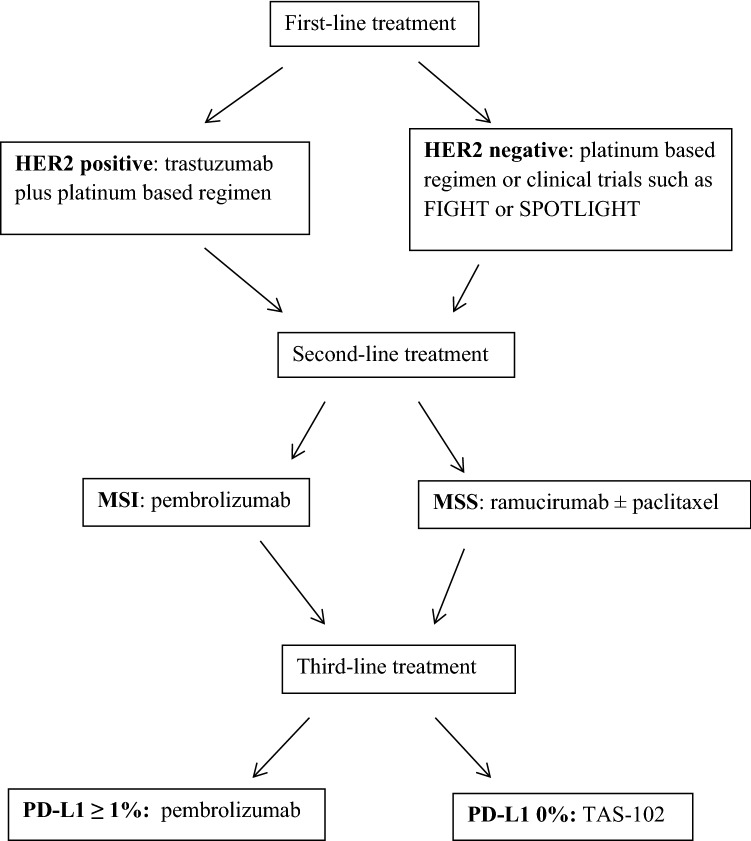
Proposed biomarker-driven algorithm for targeted and novel therapy in advanced gastric cancer

The systemic treatment options for advanced GC have evolved rapidly to incorporate targeted therapies with biomarker selection (Fig. 1). Addition of trastuzumab to platinum-based chemotherapy has become standard of care as front-line therapy in advanced GC overexpressing HER2. In the second-line setting, ramucirumab with paclitaxel for unselected population and pembrolizumab for microsatellite instability-high tumors are approved options in USA. For patients with refractory disease, apatinib, nivolumab, ramucirumab and TAS-102 have demonstrated single-agent activity with improved overall survival compared to placebo alone. Pembrolizumab is approved in tumors expressing programmed death ligand 1 as third-line treatment. Emerging data have implicated the benefit of PD-1 inhibitor in first and second-line setting. Current trials incorporating targeted therapies with chemotherapy in the front-line and adjuvant settings are ongoing.

## Data Availability

Data sharing not applicable to this article as no data sets were generated or analyzed.

## Abbreviations

GC: gastric cancer
GEJ: gastroesophageal junction
OS: overall survival
5-FU: 5-fluorouracil
PI3K/mTOR: phosphatidylinositol-3-kinase/mammalian target of rapamycin
TCGA: The Cancer Genome Atlas
EBV: Epstein–Barr virus
PIK3CA: phosphatidylinositol-4,5-bisphosphate 3-kinase catalytic subunit alpha
PD-L1/PD-L2: programmed death ligand 1/2
MSI: microsatellite instability
CIN: chromosomal instability
VEGFA: vascular endothelial growth factor A
RTK: receptor tyrosine kinase
HER2: human epidermal growth factor receptor 2
GS: genomically stable
MLH1: mutL homolog 1
MSS: microsatellite stable
EMT: epithelial-to-mesenchymal transition
MET: mesenchymal–epithelial transition
FGFR2: fibroblast growth factor receptor 2
VEGFR2: vascular endothelial growth factor receptor 2
PD-1: programmed death-1
IHC: immunohistochemistry
FISH: fluorescence in situ hybridization
PFS: progression-free survival
RR: response rate
HGF: hepatocyte growth factor
ADC: antibody–drug conjugate
ADCC: antibody dependent cell-mediated cytotoxicity
DCR: disease control rate
CR: complete response
PR: partial response
SD: stable disease
EGFR: epidermal growth factor receptor
CapeOx: capecitabine and oxaliplatin
FOLFOX: 5-FU/leucovorin/oxaliplatin
CPS: combined positive score
AE: adverse event
TP: thymidine phosphorylase
